# Whither Anal Cancer?

**DOI:** 10.1038/s41416-019-0690-4

**Published:** 2020-01-14

**Authors:** Alexander G. Heriot

**Affiliations:** 1 Division of Cancer Surgery, Peter MacCallum Cancer Centre, Melbourne, VIC Australia; 2 The Sir Peter MacCallum Department of Oncology, University of Melbourne, Melbourne, VIC Australia

**Keywords:** Drug development, Gastrointestinal cancer, Anal cancer

## Abstract

Improving outcomes for uncommon tumours such as anal cancer can be challenging. Evolution of care tends to be incremental rather than transformative and identifying the specific factors facilitating improvement can be difficult. Outcome from specialist units may be better than that demonstrated in trials, posing challenges for development of future trials.

## Main

Improving outcomes for uncommon tumours such as anal cancer can be challenging. Evolution of care tends to be incremental rather than transformative and identifying the specific factors facilitating improvement can be difficult. Outcome from specialist units may be better than that demonstrated in trials, posing challenges for development of future trials.

Anal cancer remains a rare disease, comprising less than 5% of all carcinomas of the lower gastrointestinal tract, with an annual incidence rate ranging from 1 to 1.9 per 100,000 population in Western countries.^1^ Unusually, however, the incidence of anal cancer has significantly increased over the past 30 years, tripling in Australia from 0.6 per 100,000 population to 1.8 per 100,000,^2^ and increasing by 70% in the UK since the early 1990s, with a predicted rise of a further 43% by 2035.^3^ Anal cancer mortality rates have increased 22% over the last decade in the UK, with a predicted risk of increasing by 51% by 2035.^3^

With this context, it is encouraging to read the report in the *British Journal of Cancer* by Sekhar et al.^4^ from the Christie Hospital in Manchester, reporting a striking improvement in outcome for anal cancer over the last 25 years. Loco-regional failure has been halved to 16%, overall survival increased from 60 to 76%, and cancer-specific survival increased from 62 to 80%. Identifying the cause of this improvement is more difficult; however, as unlike the situation with a number of other solid tumours, such as the introduction of neoadjuvant therapy and total mesorectal excision for rectal cancer, or breast-conserving surgery, adjuvant chemotherapy and hormonal therapy for breast cancer, there has been no dramatic change in therapeutic approach for anal cancer over this time. Treatment for anal cancer has evolved incrementally since Nigro’s introduction of definitive radiotherapy in 1973,^5^ driven by evidence provided by the six Phase 3 randomised trials for treatment of primary anal cancer. Chemoradiotherapy has been shown to be better than radiotherapy alone; the additional of mitomycin is better than 5-fluoruracil alone; cisplatin is no better than mitomycin; and there is no benefit from neoadjuvant or adjuvant chemotherapy. So why has there been such an improvement in outcome? Sekhar et al.^4^ speculate that improvements in imaging may facilitate more accurate treatment application, and it is very likely that this would also account for the almost 25% increase in node-positive tumours over the duration of their study. Refinements in radiotherapy technology and removal of inter-therapy treatment breaks are also likely strong contributors. This will also overlap with the proposed impact of centralisation of care, and the potential of experiential learning and refinement of techniques such as intensity modulated radiation therapy (IMRT) resulting in individual treatment refinement for patients. Improvement in the general health of the population may also indirectly improve outcome, as anal cancer mortality remains greater in patients from deprived areas. Arguments can be made for all these points; however, the challenge is transferring the excellent outcomes produced by this centre to the broader population when the specific changes that have resulted in this improvement are not defined.

Presentation of high-quality results from a single centre also raises a further challenge touched upon by the authors. The outcomes are better than those generated by historic trials; however, when trying to apply this to a broader context such as powering the next generation of clinical trials, what figure should be used? Application of the best reported outcomes may result in a potentially beneficial intervention failing to demonstrate a significant improvement; or application of the outcome of the most recent large multicentre trial, however, that may also potentially produce a non-significant trial if the control group of standard of care is better than expected as a result of non-specified improvements over time? The duration and cost of large scale randomised controlled trials is such that the impact of this decision can be very significant. The current recruiting anal cancer trial, the PLATO (personalizing radiotherapy dose in anal cancer) trial, has taken a very pragmatic approach. Generation of an umbrella trial comprising three separate trial, ACT 3, 4 and 5, allows three different questions to be addressed, facilitating individualisation of management.^6^ Selective application of chemoradiotherapy to early locally excised anal margin tumours (ACT3); randomising reduction of chemoradiotherapy dose for intermediate risk tumours to reduce late effect complications (ACT 4); and randomising chemoradiotherapy dose escalation for locally advanced tumours (ACT 5), will allow the spectrum of anal cancers to be assessed within the same trial and the results will be eagerly awaited.^7^

Changes in management of anal cancer have been gradual and incremental with an 16% improvement in survival over 25 years so is there the potential for any disruptive change in management going forward in an orphan tumour? Immunotherapeutic strategies have become a focus of research efforts for anal cancer, with the impact of the human papilloma virus on the oncogenesis of anal cancer an important consideration.^8^ A recent Phase 2 study of nivolumab, an anti-PD1 antibody, has demonstrated positive results in the management of metastatic anal cancer.^9^ Potential roles in primary disease are yet to be explored but with increasing interest in the molecular drivers of anal cancer, the future is exciting and timely, as despite treatment getting better, anal cancer is becoming more common.^10^

## Data Availability

Not applicable.
